# The Study of Chromobox Protein Homolog 4 in 3D Organoid Models of Colon Cancer as a Potential Predictive Marker

**DOI:** 10.3390/ijms26157385

**Published:** 2025-07-30

**Authors:** Vincenza Ciaramella, Valentina Belli, Francesco Izzo, Andrea Belli, Antonio Avallone, Alfonso De Stefano, Andrea Soricelli, Anna Maria Grimaldi

**Affiliations:** 1 IRCCS SYNLAB SDN, 80143 Naples, Italy; vincenza.ciaramella@synlab.it (V.C.); andrea.soricelli@synlab.it (A.S.); 2 Unit of Hepato-Biliary and Pancreatic Surgery, Istituto Nazionale Tumori IRCCS Fondazione G. Pascale, 80131 Naples, Italy; valentina.belli@istitutotumori.na.it (V.B.); f.izzo@istitutotumori.na.it (F.I.); a.belli@istitutotumori.na.it (A.B.); 3 Experimental Clinical Abdominal Oncology Unit, Istituto Nazionale Tumori IRCCS Fondazione G. Pascale, 80131 Naples, Italy; a.avallone@istitutotumori.na.it (A.A.); a.destefano@istitutotumori.na.it (A.D.S.); 4 Department of Medical, Movement and Well-being Sciences, University of Naples Parthenope, 80133 Naples, Italy

**Keywords:** CBXs, colorectal cancer, organoids, prognostic factors, biomarkers

## Abstract

The Chromobox (CBX) family comprises key epigenetic regulators involved in transcriptional repression through chromatin modifications. Dysregulation of polycomb CBX proteins has been linked to epigenetic gene silencing and cancer progression. However, the specific roles and prognostic value of CBX family members in colorectal cancer (CC) remain unclear. In this study, we show that CBX genes are significantly dysregulated in CC tissues and cell models compared to normal colorectal tissue. Among them, CBX4 and CBX8 emerged as the most upregulated isoforms in tumors. Functional analyses revealed that CBX4 overexpression enhances CC cell proliferation, while its silencing reduces tumor growth. Similarly, pharmacological inhibition of CBX4 in patient-derived tumor organoids led to decreased proliferation, supporting its pro-tumorigenic role. Immunofluorescence analysis further revealed alterations in NF-κB signaling upon CBX4 inhibition, along with reduced mRNA levels of pathway components including NF-κB, TNF, IL-1, and c-Myc. These findings point to a potential interplay between CBX4 and inflammation-related pathways in CC. Overall, our study highlights the oncogenic role of CBX4 in colorectal cancer and supports its potential as a novel therapeutic target and early biomarker for disease progression.

## 1. Introduction

Chromobox family (CBX) proteins, known as integral components of polycomb group complexes, play crucial roles in numerous biological processes such as embryonic development, stem cell maintenance, cell proliferation, and apoptosis [1]. Increasing evidence suggests their involvement in regulating tumor-related processes like cell cycle [2], chemotherapy and radiotherapy sensitivity [3], tumor cell stemness [4], and metabolism [5], affecting tumor initiation and progression significantly [6,7].

The CBX family can be categorized into two subgroups based on their molecular structure: the HP1 group (including CBX1, CBX3, and CBX5) and the Pc group (including CBX2, CBX4, CBX6, CBX7, and CBX8). While some CBX genes and proteins have been studied in relation to cancers like breast, liver, and gastric cancer, the roles of CBX family members in colorectal cancer (CRC) are still unclear [8]. The biological functions and prognostic significance of CBX1-8 in cancer progression remain uncertain, with misregulation of CBX proteins being linked to various cancer types. Further research is needed to determine the specific functions and prognostic values of different CBX family members in CRC.

Colorectal cancer (CRC) is the third most prevalent form of cancer worldwide and the second leading cause of cancer-related mortality [9]. Its incidence is rising among the young [10]. Guidelines now recommend screening for colon cancer at age 45 [11]. Circulating tumor DNA can be used to select patients for adjuvant chemotherapy. Immunotherapy is an option for patients with mismatch repair protein deficiencies, but its efficacy outside of this group remains unclear. Targeted therapies such as BRAF inhibitors are an option for patients with poor prognoses, for whom chemotherapy is ineffective [12].

Understanding the molecular mechanisms through which CBX proteins modulate tumorigenesis may reveal novel therapeutic targets for cancer intervention. Several chromatin-associated regulatory proteins have already been identified as candidate targets for anti-cancer therapy, with small-molecule inhibitors currently in development or undergoing clinical trials. This underscores the increasing scientific and clinical interest in CBX proteins and their associated signaling pathways as viable targets in oncology. In this context, we expanded our investigation to CRC, utilizing multiple large-scale datasets to assess the potential oncogenic roles of individual CBX family members in CRC. Moreover, we aimed to deepen the role of CBX4 in CRC. Among the CBX family members, CBX4 stands out due to its dual functionality, combining both epigenetic and post-translational regulatory roles [13]. CBX4 has been implicated in tumor progression in hepatocellular [14,15], gastric [4], and lung cancers [16,17] through mechanisms involving angiogenesis, SUMOylation, and epigenetic repression. However, despite growing interest in CBX proteins as key players in cancer biology, the role of CBX4 in CRC remains poorly investigated, particularly in relation to its potential involvement in inflammatory signaling pathways, which are central to colorectal tumorigenesis. Therefore, we focused our study on CBX4, characterizing its expression profile, regulatory functions, and clinical significance in CRC. By integrating analyses of clinical samples, in vitro cell models, and patient-derived organoids, we also linked CBX4 regulators to inflammation-driven tumor progression in CRC.

## 2. Results

### 2.1. Characterization of CBX Family in CRC Cell Lines and Patients

**Figure 1:**
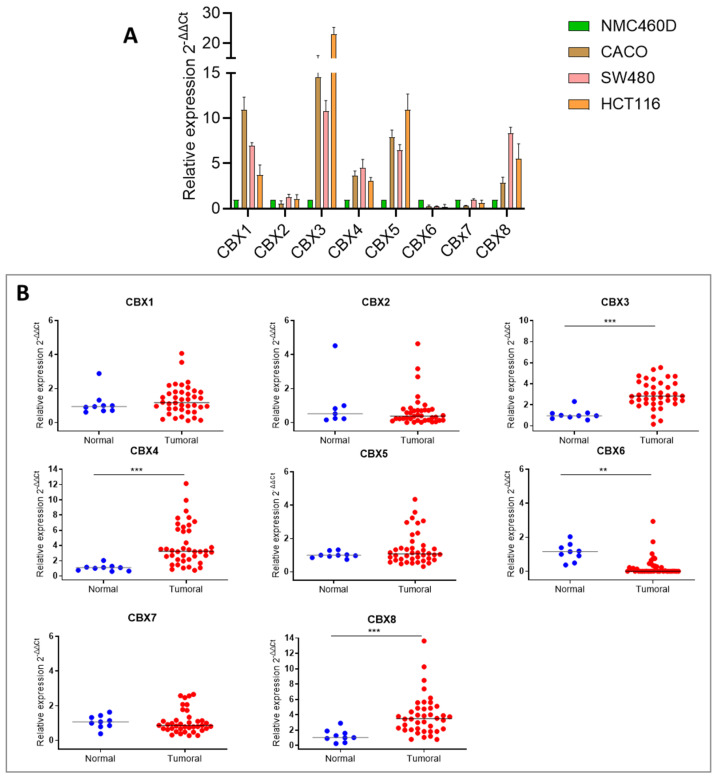

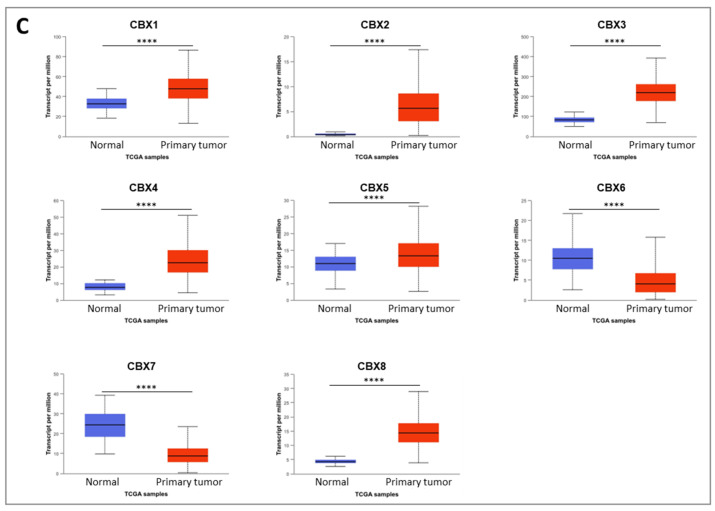
CBX family genes expression in colon cancer. (**A**) CBX expression level by qRT-PCR in three colon cancer cell lines (CACO-2, SW480, and HCT 116) compared to a normal colon cell line (NMC 460D). (**B**) CBX gene expression level in a colon cancer cDNA RT-qPCR array consisting of tumor (*N* = 40) and normal samples (*N* = 8). (**C**) Expression analysis of CBX family genes of colon tumor in UALCAN dataset (Normal = 41, Primary Tumor = 286). The star symbol indicates statistical significance (**: *p* ≤ 0.01, ***: *p* ≤ 0.001, ****: *p* ≤ 0.0001).

As a first step, we evaluated the expression levels of the CBX family members (CBX1–8) in CRC cell lines (HCT116, SW480, and CACO-2) compared to the non-cancerous colonic epithelial cell line NCM 460D. As shown in Figure 1A, most CBX genes were upregulated in CRC cell lines. Notably, CBX6 and CBX7 were the only members lower expressed across all CRC cell lines assessed.

To validate these findings in clinical samples, we next evaluated CBX genes expression in colorectal tissue specimens from healthy and tumor patients using TissueScan qPCR Arrays (Figure 1B). A significant dysregulation was observed for CBX3, CBX4, and CBX8, all of which were upregulated in tumor tissues. Conversely, CBX6 consistently exhibited significant downregulation in tumor tissues. The expression of other CBXs in colon cancer patients, although not displaying a significant dysregulated profile, confirmed the pattern observed in cell lines. Given the limited size of our patient cohort, we extended our analysis using the UALCAN database, which includes TCGA transcriptomic data from a larger number of CRC samples. As shown in Figure 1C, the expression trends observed in our experimental analyses were largely confirmed, with greater statistical power, likely due to the larger sample size dataset.

**Figure 2:**
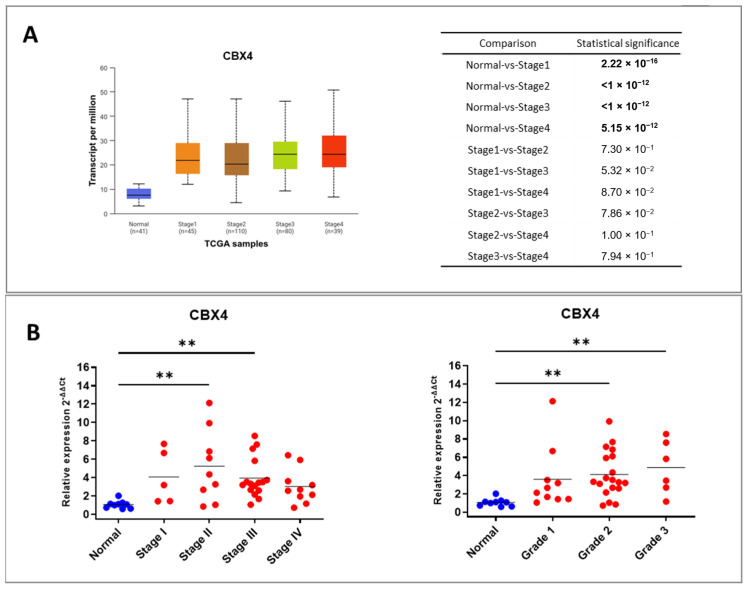
Association between CBX4 expression levels and clinical stage and grade of CRC patients. (**A**) CBX4 expression analyses performed using the UALCAN dataset with relative statistical comparison among groups. (**B**) CBX4 expression analyses in a colon cancer cDNA RT-qPCR array consisting of tumor (*n* = 40) and normal samples (*n* = 8) stratifying patients according to stage and grade. The star symbol indicates statistical significance (**: *p* ≤ 0.01).

Among the dysregulated CBX genes, CBX4 emerged as particularly interesting due to its limited characterization in colorectal cancer. To further explore the clinical relevance of CBX4, we examined its expression in relation to tumor stage using the UALCAN TCGA dataset (Figure 2A). Patients showed elevated CBX4 levels across all cancer stages (I–IV), with no significant variation among stages, suggesting that CBX4 overexpression may occur early in tumor development and persist during disease progression. These findings were confirmed in an independent cohort of colorectal cancer tissues, where CBX4 expression remained elevated across stages, particularly in stages II and III (Figure 2B), and across tumor grades, particularly in grades II and III (Figure 2B). Although some intra-group variability was observed, the overall trend supports the robust overexpression of CBX4 in CRC, regardless of tumor grade or stage. Similar expression trends for the other CBX family members were also observed across both the PCR array dataset and the TCGA-UALCAN analysis. For a more comprehensive overview, we refer the reader to Supplementary Figures S1–S4.

### 2.2. Effect of CBX4 Knockout in CRC Cell Lines

**Figure 3:**
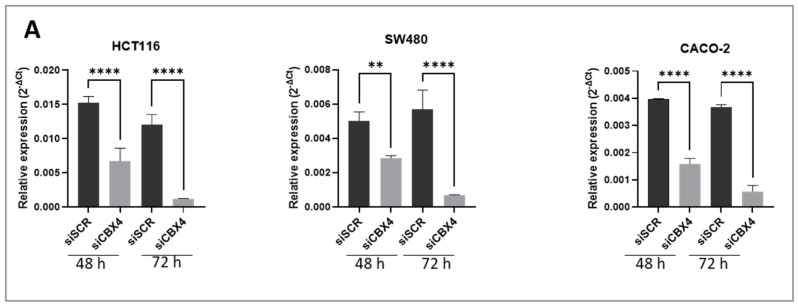

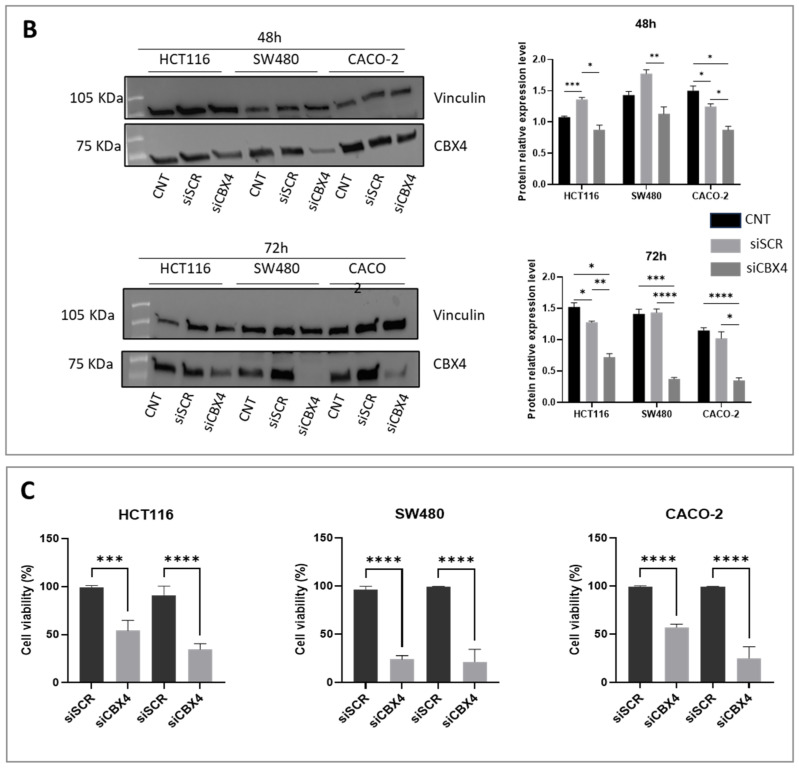
Knockdown efficiency of siCBX4. (**A**) Analysis of the knockdown efficiency of siCBX4 assessed by RT-PCR in three colon cancer cell lines after 48 h and 72 h. (**B**) Analysis of the knockdown efficiency of siCBX4 assessed by Western blot after 48 h and 72 h, with relative densitometry of CBX4 protein expression. (**C**) Effect of CBX4 silencing on cell viability assessed by ATP Lite assay after 48 h and 72 h. The star symbol indicates statistical significance (*: *p* ≤ 0.05, **: *p* ≤ 0.01, ***: *p* ≤ 0.001, ****: *p* ≤ 0.0001).

**Figure 4:**
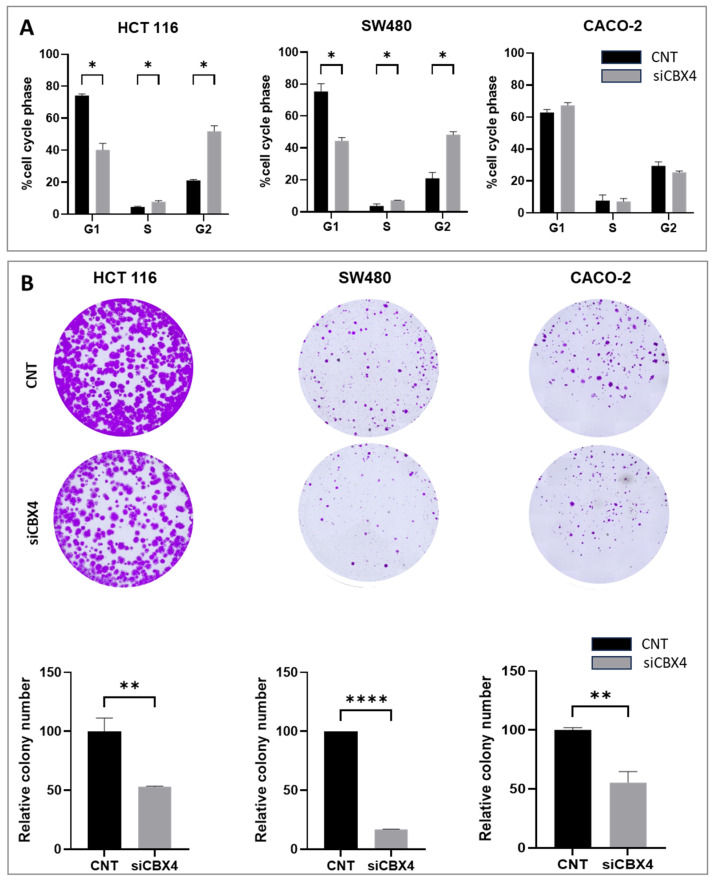
Effect of CBX4 silencing on colorectal cancer cell proliferation. (**A**) Flow cytometry analysis of HCT116, SW480, and CACO-2 cell cycle and relative % (*: *p* < 0.05). (**B**) Colony formation of CBX4 knockdown and untreated group cells was photographed, and colony numbers were illustrated in histogram. The star symbol indicates statistical significance (*: *p* ≤ 0.05, **: *p* ≤ 0.01, ****: *p* ≤ 0.0001).

To investigate the functional role of CBX4 in CRC colorectal cancer (CRC), we performed transient silencing using siRNA in three CRC cell lines: HCT116, CACO-2, and SW480. To set up siRNA knockdown experiments, four siRNAs against CBX4 (siCBX4_s1, siCBX4_s2, siCBX4_s3, and siCBX4_s5) were transfected into HCT116 cell line, chosen due to its high expression of protein CBX4. Through Western blot analysis (Supplementary Figure S1), we found that siAURKA-s5 had the strongest knockdown effect compared to the Scramble sequence (siSCR), indicating that this silencing experiment was successful. Based on these results, siCBX4-s5 was selected for all subsequent gene silencing experiments presented in this study. Efficient knockdown of CBX4 was confirmed at both mRNA and protein levels at 48 and 72 h post-transfection in all three cell models (Figure 3A,B). A slight variation in CBX4 expression was observed between 48 h and 72 h in the siSCR group, likely reflecting minor biological variability. However, this did not impact the overall trend. Notably, at 72 h, CBX4 expression was consistently and significantly reduced in siCBX4-treated cells compared to both control and scrambled siRNA groups, confirming the effectiveness of the silencing (Figure 3B). Silencing of CBX4 resulted in a significant reduction in cell proliferation compared to control cells (Figure 3C), as assessed by ATP Lite assay. Cell cycle analysis by flow cytometry revealed an accumulation of cells in the G2 phase following CBX4 knockdown (Figure 4A), suggesting a block in cell cycle progression. This G2 arrest was consistently observed across two of three cell lines, HCT116 and SW480 at 72 h post-silencing, whereas no change was detectable in CACO-2 cells. However, colony formation assays demonstrated a marked decrease in the number and size of colonies in all CBX4-silenced cells (Figure 4B), indicating impaired clonogenic potential. Collectively, these results indicate that CBX4 contributes to CRC cell proliferation and cell cycle progression, and its silencing impairs tumorigenic potential by inducing G2 phase arrest and reducing long-term proliferative capacity.

### 2.3. Characterization of CBX4 in Patient-Derived Organoids of Colorectal Cancer

**Figure 5:**
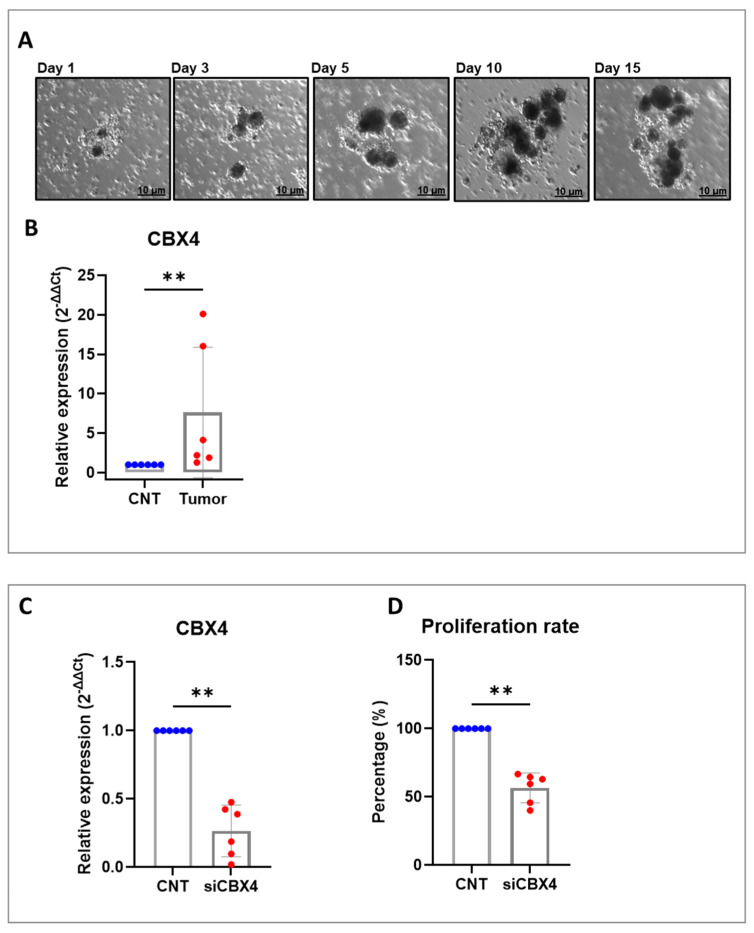
Characterization of CBX4 in CRC organoids. (**A**) Representative images of ex vivo PDO culture obtained from colorectal cancer biopsies are reported. Scale bar: 10 µm. (**B**) Real-time qPCR analysis of CBX4 in two groups of PDOs: the first one derived from healthy tissues (CNT) and the second one derived from tumor tissues (Tumor). Results were normalized to RPS18 mRNA and analyzed by 2^−ΔΔCt^ method. (**C**) Analysis of the knockdown efficiency of siCBX4 assessed by RT-PCR in PDOs after 72 h. (**D**) Effect of CBX4 silencing on PDO viability assessed by ATP Lite assay after 72 h. The star symbol indicates statistical significance (**: *p* ≤ 0.01).

To evaluate the feasibility and comparative utility of both in vitro and ex vivo models for colorectal cancer research, we established 3D culture systems using freshly resected surgical tumor specimens. Specifically, ex vivo cultures were developed through enzymatic dissociation of human colon tumor tissues, enabling the generation of PDOs as primary cell cultures (Figure 5A). This approach allowed for the rapid establishment of organotypic spheroid cultures that closely mimic the architecture and cellular heterogeneity of the original tumors. The resulting ex vivo PDOs consist of multicellular aggregates that not only maintain key intercellular interactions but also recapitulate critical components of the tumor microenvironment, including epithelial, stromal, and a part of immune cell populations. Importantly, these cultures retain the genetic diversity and clonal composition observed in vivo, making them highly relevant for studying tumor biology, drug response, and resistance mechanisms in a personalized context. To investigate the clinical relevance of our model, we analyzed 12 samples of human PDOs: 6 samples derived from colorectal cancer tissues and a control group comprising 6 samples derived from healthy colon sections. In this initial analysis, we observed a significantly elevated expression of CBX4 mRNA in all tumor-derived PDOs compared to the healthy controls, indicating a tumor-specific upregulation of this gene (Figure 5B). To corroborate the findings obtained in established CRC cell lines and to enhance the translational relevance of our study, we extended our experimental approach to include patient-derived CRC organoids. Notably, we successfully achieved transient silencing of CBX4 using siRNA, allowing us to investigate its functional role in CRC. Efficient knockdown of CBX4 was confirmed at the mRNA level 72 h post-transfection (Figure 5C). Moreover, proliferation assays revealed that siCBX4 resulted in a significant reduction in cell proliferation compared to the control group (Figure 5D), underscoring the potential oncogenic role of CBX4 in CRC pathobiology.

### 2.4. CBX4 Knockdown Reduces NF-κB Activation in CRC Organoids

To investigate the involvement of transcriptional regulators in CRC progression, we assessed the role of NF-κB, a transcription factor known to be constitutively activated in various malignancies due to autocrine and paracrine signaling within the tumor microenvironment.

**Figure 6:**
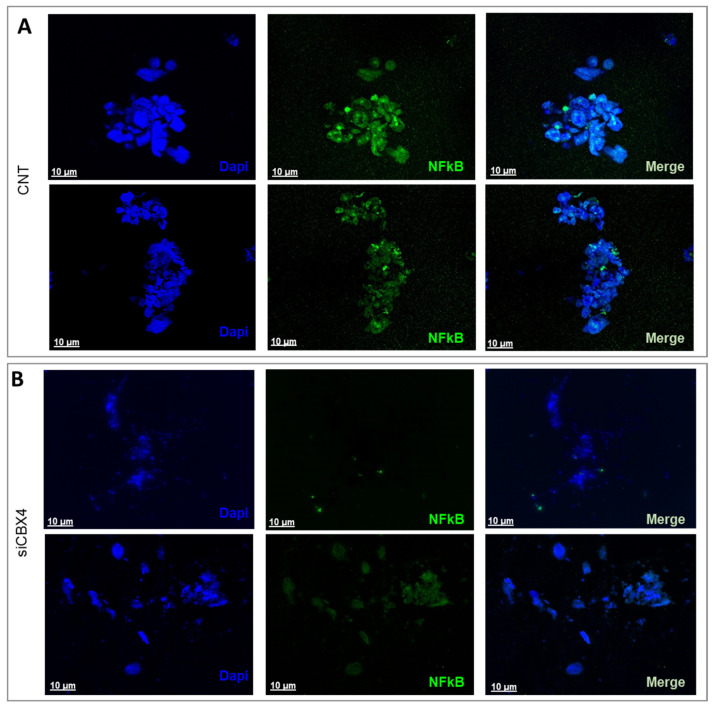

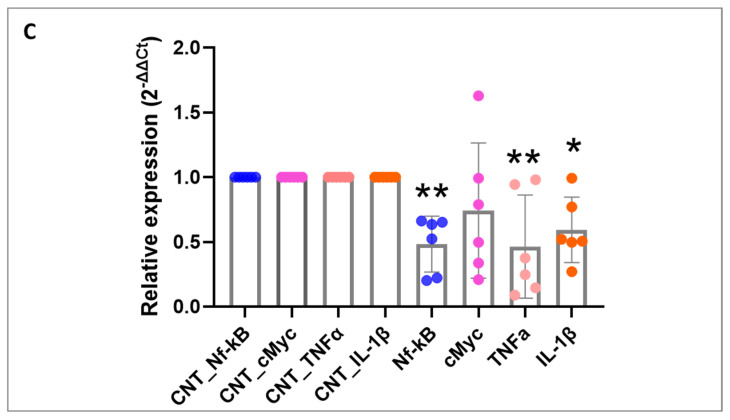
Role of CBX4 silencing in CRC organoids (**A**,**B**). Representative images of our target molecules through immunofluorescence analysis. Localization of NFkB in tumor ex vivo PDOs before (CNT) and after siCBX4. Maximal projection images of PDO incubated NFkB (green signal) and cell nuclei were stained with Hoechst 33342 (blue signal). Scale bar: 10 µm. (**C**) Real time qPCR analysis of NF-κB, c-myc, TNF-α, and IL-1 in tumor ex vivo PDOs before (CTR) and after CBX4 silencing. Results were normalized to RPS18 mRNA and analyzed by 2^−ΔΔCt^ method. The star symbol indicates statistical significance (*: *p* ≤ 0.05, **: *p* ≤ 0.01).

Immunofluorescence analysis was conducted to evaluate NF-κB expression in 3D PDO models of CRC. In untreated PDOs, NF-κB displayed strong nuclear localization (Figure 6A). Following siCBX4, a substantial reduction in NF-κB signal was observed (Figure 6B), indicating that CBX4 may positively regulate NF-κB activity. To validate this observation at the transcriptional level, qPCR analysis was performed to measure NF-κB mRNA expression. A significant downregulation of NF-κB transcripts was detected in CBX4-silenced samples compared to controls. Given that NF-κB activation can be driven by upstream factors such as the pro-inflammatory cytokines TNF-α and IL-1, as well as the oncogene c-Myc, we also quantified the expression of these genes. CBX4 knockdown resulted in reduced expression of TNF-α, IL-1, and c-Myc, suggesting that CBX4 may regulate NF-κB activation through modulation of its upstream signaling components (Figure 6C).

## 3. Discussion

CBXs form a family of eight proteins that are integral components of polycomb group complexes and play a crucial role in epigenetic regulation, gene silencing, and chromatin remodeling [18]. In this study, we first provide a comprehensive analysis of the expression patterns and functional relevance of CBX proteins in CRC.

Our findings show that CBX3, CBX4, and CBX8, all members of the polycomb (Pc) group of the non-canonical PRC1 (nPRC1) complex, are significantly upregulated in both CRC cell lines and tumor tissues. In contrast, CBX6 displays a consistent downregulation across both CRC cell lines and tumor samples. The role of this gene appears highly context-dependent, acting either as an oncogene or tumor suppressor depending on the cancer type and cellular environment. For instance, significant downregulation of CBX6 was reported in breast cancer, where its ectopic overexpression inhibited tumor progression [19]. Otherwise, in liver cancer, elevated CBX6 expression was associated with a worse prognosis [13]. The consistent downregulation of CBX6 observed in our study supports previous pan-cancer analyses of CBX gene expression and prognosis [20]. It suggests a tumor-suppressive role for CBX6 in colorectal cancer, particularly in COAD.

Notably, CBX4 has been reported to play a pivotal role in epigenetic regulation, contributing to transcriptional repression and the DNA damage response through its chromatin-binding ability and SUMO E3 ligase activity [21], influencing the transcriptional activity of critical transcription factors and controlling the cell cycle in cancer cells [22]. It is involved in various biological processes, including cell growth, senescence, and cancer development. Its expression levels influence tumor initiation and patient prognosis across multiple cancer types [23]. Several studies have demonstrated that CBX4 expression scores are significantly higher in the recurrence group compared to the non-recurrence group, suggesting that elevated CBX4 expression may be associated with poor prognosis in patients with colon cancer. Moreover, CBX4 expression has shown a positive correlation with postoperative recurrence in early-stage colon cancer cases [24]. However, the role of CBX4 in CRC remains understudied: its function in CRC and its possible interaction with inflammatory pathways—central to colorectal tumorigenesis—have not been systematically investigated. Given this uncertainty and the increasing evidence linking CBX proteins to inflammation-driven cancer progression, we focused our study on CBX4 to elucidate its expression pattern, regulatory networks, and clinical relevance in CRC.

Functional assays following CBX4 knockdown in colon cancer cells demonstrated that its silencing leads to a marked reduction in cell proliferation, induction of G2 cell cycle arrest, and impaired clonogenic potential, underscoring its contribution to tumor cell growth and survival. These results are in line with previous findings in other cancer types, where CBX4 has been implicated in promoting oncogenic behaviors via epigenetic mechanisms. Interestingly, although CBX4 has been reported to act as a tumor suppressor by interacting with histone deacetylase 3 (HDAC3) to inhibit RUNX2 gene expression [25], our data suggest that CBX4 may also function as an oncogenic driver in a broader cellular context, supporting CRC cell proliferation and tumorigenic capacity. This apparent dual role may reflect a context-dependent function of CBX4, influenced by the specific epigenetic landscape or interaction partners in different cellular environments.

By integrating in vitro assays with ex vivo tumor organoid cultures, we aimed to develop a complementary platform for translational colorectal cancer research. This dual approach enhances our ability to study tumor behavior under controlled conditions while preserving the complex interactions within the native microenvironment. Organoids, derived directly from patient tissues, closely mimic the in vivo clinical setting by retaining the unique genetic, molecular, and cellular characteristics of the original tumor, including its heterogeneity and microenvironmental features. Compared to traditional cell lines, they offer a more physiologically accurate model for investigating disease progression, drug responses, and therapeutic interventions. This patient-specific platform enables diverse applications such as studying tumor-immune interactions, identifying novel molecules involved in immunotherapy response, and predicting clinical outcomes in a reproducible, straightforward, and cost-effective manner. Overall, these 3D patient-derived models are valuable tools for pre-clinical testing, biomarker discovery, and developing personalized therapeutic strategies. Depending on the cell type, NF-κB is most potently activated following engagement with pattern-recognition receptors, receptors for pro-inflammatory cytokines such as TNF-α or IL-1, and antigen receptors [26]. These cytokines regulate and mediate the crosstalk between tumor cells and tumor-infiltrating immune and inflammatory cells, and they are responsible both for inflammation-driven tumor growth and the inhibition of antitumor immune surveillance. It is important to highlight that, although cytokines in the tumor microenvironment are mainly produced by hematopoietic cells, some of them can also be produced directly by malignant tumor cells to establish an autocrine pro-tumor signaling loop that further enhances NF-κB activation [27]. In our study, we demonstrated that silencing CBX4 in patient-derived organoids leads to NF-κB inactivation and a consequent reduction in the expression of molecules involved in this activation, such as TNF-α and IL-1. This finding suggests that CBX4 may regulate the expression of genes involved in cell proliferation and survival, both in premalignant cells and their neoplastic progeny, through the NF-κB signaling circuit, potentially serving as an early disease marker.

Concluding, our findings highlight CBX4 as a key epigenetic regulator in CRC. Its consistent overexpression in cell lines, tumor tissues, and patient-derived organoids, along with its role in promoting proliferation and sustaining NF-κB–mediated inflammatory signaling, suggests a pro-tumorigenic function. siCBX4 impaired CRC cell growth and reduced the expression of key oncogenic and inflammatory markers. These results support CBX4 as a potential therapeutic target and early biomarker in CRC. Further investigations are warranted to dissect the molecular mechanisms underlying CBX4-mediated oncogenic activity in CRC and to determine whether its inhibition could be leveraged therapeutically, either alone or in combination with existing treatment strategies.

Given its central role in repressing tumor suppressor genes and promoting oncogenic pathways, CBX4 represents a promising target for therapeutic intervention in CRC. Its inhibition has been shown to reduce tumor cell proliferation and enhance sensitivity to treatment. Furthermore, the detection of CBX4 overexpression in early tumorigenesis suggests its potential as an early diagnostic biomarker [28]. Recent evidence also implicates CBX4 in chemotherapy resistance, particularly through the regulation of YAP1 SUMOylation and the suppression of cellular senescence [29]. Future studies will focus on elucidating CBX4′s role in treatment resistance and its clinical utility as both a therapeutic target and a biomarker to guide personalized treatment strategies.

Future studies may investigate the potential of combining CBX4 inhibitors with immune checkpoint inhibitors as a novel therapeutic strategy. Given CBX4′s role in modulating gene expression, chemoresistance, and possibly immune evasion, its inhibition could enhance the efficacy of immunotherapy by promoting a more immunogenic tumor microenvironment. Specifically, CBX4 blockade may facilitate T cell infiltration and activation, thereby synergizing with checkpoint blockade therapies such as anti-PD-1 or anti-CTLA-4. Preclinical models will be essential to assess the therapeutic benefit, identify predictive biomarkers, and optimize treatment regimens, ultimately paving the way for translational applications in CRC.

## 4. Materials and Methods

### 4.1. CBX4 Knockdown Reduces NF-κB Activation in CRC Organoids

The human colon cancer cell lines CACO-2 (DMSZ #ACC 169), HCT 116 (DMSZ #ACC 581), and SW480 (DMSZ #ACC 313), as well as the human normal epithelial cell line NMC 460D (INCELL, San Antonio, TX, USA), were cultured according to the manufacturer’s recommendations. CACO-2 cells were grown in 80% MEM (with Earle’s salts) supplemented with 20% HI-FBS and 1× non-essential amino acids. HCT 116 cells were cultured in 90% McCoy’s 5A medium, supplemented with 10% heat-inactivated (hi) FBS and 2 mM L-glutamine. SW480 cells were grown in 90% RPMI 1640, supplemented with 10% hi-FBS. The NMC 460D cells were grown in DMEM high glucose with 10% hi-FBS and 2 mM L-glutamine. The cells were seeded at approximately 1 × 10^6^ cells/80 cm^2^, split when the culture reached confluence, every 3–4 days using trypsin/EDTA. The cells were maintained at 37 °C in a humidified chamber at 5% CO_2_. Cell stocks were prepared by cryopreserving cells in a solution of 90% FBS and 10% dimethyl sulfoxide (DMSO; Sigma-Aldrich, Merck, Darmstadt, Germany) and stored either at −80 °C for up to 30 days or in liquid nitrogen for long-term preservation.

### 4.2. Transfection of Small Interfering RNA (siRNA)

CBX4 siRNA and negative control siRNA were purchased from Qiagen (GS8535—FlexiTube GeneSolution GS8535, Hilden, Germany) to silence CBX4 expression in colon cancer cells and PDOs. It is worth noting that the siRNA sequence used for transfection was siCBX4_s5, because this sequence has previously been shown to provide the most effective silencing results. The cells and PDOs were seeded into six-well plates at 2.0 × 10^5^ cells per well. On the following day, CBX4 siRNA (siCBX4) or siRNA control (siSCR) was transfected into cells using Lipofectamine RNAimax (Thermo Fisher Scientific, Waltham, MA, USA, 13778100) according to the manufacturer’s instructions. Briefly, for each well of a 6-well plate, 30 pmol of siRNA was diluted in 150 μL of Opti-MEM without serum. Separately, 9 μL of Lipofectamine RNAiMAX was diluted in an additional 150 μL of Opti-MEM. The two solutions were mixed gently and incubated at room temperature for 10 min to allow complex formation. Then, 250 uL of resulting siRNA–Lipofectamine™ complexes was added to each well containing 1000 μL of complete growth medium and adherent cells at ~70–80% confluency. Cells were incubated at 37 °C with 5% CO_2_ for 48 to 72 h prior to subsequent analysis. Knockdown efficiency was confirmed by quantitative polymerase chain reaction (RT-qPCR) and Western blotting.

### 4.3. RNA Isolation and qRT-PCR

Total RNA was isolated from cells and PDOs using TRIzol reagent (Cat: # 15596026, Invitrogen, Carlsbad, CA, USA), followed by phase separation with chloroform and RNA precipitation with isopropanol. The RNA pellet was washed with 70% ethanol, air-dried, and resuspended in RNase-free water. RNA yield and purity were assessed using a NanoDrop spectrophotometer (Thermo Fisher). For cDNA synthesis, 1 µg of total RNA was reverse-transcribed using the SensiFAST cDNA synthesis kit (BIO-65053, Meridian Bioscience, Memphis, TN, USA), following the manufacturer’s instructions. Quantitative real-time PCR (RT-qPCR) was then performed to assess gene expression levels, and amplification was conducted using SYBR Green PCR Master Mix (Applied Biosystems by Life Technologies, Monza, Italy). The thermal cycling protocol included an initial step at 50 °C for 2 min, and denaturation at 95 °C for 10 min, followed by 40 amplification cycles of 95 °C for 15 s and 60 °C for 1 min. Reactions were set up in 20 µL volumes and run in duplicate using the QuantStudio 7 Flex Real-Time PCR System (Applied Biosystems). Relative gene expression was normalized to GADPH for cells and to 18S ribosomal RNA subunit for organoids as the endogenous control, and data were analyzed using the 2^−ΔΔCt^ method. Primer sequences are listed in Table S1. Samples from human colon carcinoma patients were obtained from commercial arrays (TissueScan™ cDNA arrays, HCRT304, OriGene, Rockville, MD, USA). Human TissueScan Colon Cancer Tissue qPCR Panel IV (HCRT304), containing first-strand cDNA from 48 samples covering 8-normal, 5-Stage I, 8-IIA, 1-II, 1-IIIA, 6-IIIB, 3-IIIC, 6-III, and 10-IV patients, was purchased from Origene (Rockville, MD, USA). Nine identical plates were used to determine the expressions of CBX1-8 and β-Actin (housekeeping gene). RT-qPCR was used to determine the condition. Data were expressed as mean fold-change using the comparative 2^−ΔΔCq^ method (compared to non-malignant control tissue).

### 4.4. Protein Extraction and Western Blot Analysis

Cells were washed twice in ice-cold PBS and lysed in JS buffer (50 mmol/L HEPES (pH 7.5) containing 150 mmol/L NaCl, 1% glycerol, 1% Triton X100, 1.5 mmol/L MgCl_2_, 5 mmol/L EGTA, 1 mmol/L Na_3_VO_4_, and 1× protease inhibitor cocktail). Protein concentration was determined by Bradford assay (Bio-Rad, Hercules, CA, USA), and equal amounts of proteins were analyzed by SDS-PAGE (TGX Stain-Free Precast Gels (8/16%), Biorad, Hercules, CA, USA). Gels were electroblotted onto polyvinylidene difluoride membranes (Millipore). For immunoblot experiments, membranes were blocked for 1 h with 5% nonfat dry milk in TBS containing 0.1% Tween 20 and incubated at 4 °C overnight with primary antibody: anti-CBX4 (1:1000; # mAb #30559, Cell Signaling Technology, Danvers, MA, USA) and anti-vinculin (1:2000; Cat: # V9264, Sigma-Aldrich). After washing with Tris-buffered saline with 0.1% Tween-20, blots were incubated with the appropriate secondary antibodies for 1 h at room temperature and developed using ECL detection reagent.

### 4.5. Cell Viability Assay

An ATP-based luminescence assay (ATP Lite 1-step Luminescence Assay System, Revvity) was performed to quantitatively assess cell proliferation and cytotoxicity in cultured cells. Briefly, cells and PDOs were seeded into white-walled 96-well plates and treated according to the experimental protocol. On the day of analysis, the culture medium was aspirated, and luminescence detection reagent was added directly to each well to induce cell lysis. The plates were then incubated at room temperature for 10 min to ensure complete lysis and stabilization of the luminescent signal. Luminescence was measured using a Victor Nivo Multimode Microplate Reader (PerkinElmer, Waltham, MA, USA) at a detection wavelength of 560 nm. Signal intensities were normalized to internal controls, and results were expressed in relative luminescence units to evaluate cell viability and proliferative capacity.

### 4.6. Colony Formation Assay

Forty-eight hours after the transfection, 5 × 10^3^ CACO-2 cells/well, 3 × 10^3^ SW480 cells/well, or 2 × 10^3^ HCT116 cells/well were seeded in six-well plates. The medium was changed every 3 days. Cells cultured for 15 days were washed twice with ice-cold medium, fixed by in 10% paraformaldehyde, and stained with crystal violet for 15 min (Sigma-Aldrich, Germany). Image acquisition was performed using a Leica microscope (Mica, Leica Microsystems, Wetzlar, Germany).

### 4.7. Patient Sample and 3D Organoid Generation

Apoptotic cells were identified using the Annexin V-FITC/AAD Kit (Beckman–Colter). The PBS-washed cells were resuspended in ice-cold 1X Binding Buffer and incubated with Annexin V-FITC and 7-amino-actinomycin D (7-AAD) on ice for 15 min in the dark, according to the manufacturer’s instructions. All samples were analyzed within 30 min by flow cytometry (Cytoflex V2-B4-R2—Beckman Colter, Brea, CA, USA). Twenty thousand events were acquired per sample. The COULTER DNA (Beckman–Coulter) Prep kit was used to assess the cell cycle of human colon cancer cells. The DNA PREP LPR reagent was used to permeabilize the cells, and DNA staining solution containing propidium iodide (PI) was used to stain DNA content. After PI staining, the quantification of the cell-cycle distribution was carried out by flow cytometry. Cell-cycle analysis was performed using the J.V. Watson algorithm. All data were analyzed using Kaluza analysis software (Beckman–Colter, CA, USA) https://www.beckman.com/flow-cytometry/software/kaluza, accessed on 1 July 2025.

### 4.8. Patient Sample and 3D Organoid Generation

To derive ex vivo 3D organoid cultures from colon cancer patient samples, we adopted a previously published protocol [30,31].

Based on the Declaration of Helsinki [32], all human samples were taken only after each patient and healthy donor provided written informed consent. All the procedures listed below were carried out in compliance with the applicable rules and laws. The study was approved by the Ethics Committee of IRCCS Pascale (Naples, Italy) with reference number 40/11 approved on 16 November 2011. Briefly, all fresh tumor tissue samples were kept on ice and processed in sterile conditions on the day of collection. Tissue fragments were digested in PDOs medium containing Hyaluronidase (Sigma-Aldrich) and Collagenase (Sigma-Aldrich) onto a 37 °C shaker at low to moderate speed (e.g., 200 rpm) for an incubation time between 12 and 18 h. Next, cells were filtered and separated with serial centrifugation. Finally, for 3D culture aggregation, cells were centrifuged, resuspended in PDO medium, and seeded in plates (Corning, New York, NY, USA). After 48 h, PDOs were visible in the cell culture plates.

### 4.9. Immunofluorescence

To characterize NF-κB expression in both tumor-derived and non-cancerous ex vivo PDOs, the organoids were incubated with a primary antibody targeting NF-κB (catalog No. 3865015; Sony Biotechnology, Inc.) at a 1:100 dilution for 1 h at room temperature. This was followed by incubation with a PE-conjugated secondary antibody (catalog No. 3D6C02; Biolegend Inc., San Diego, CA, USA) at a 1:200 dilution for 1 h at RT, protected from light. After antibody staining, the samples underwent three washing steps, were counterstained with DAPI (Sigma-Aldrich) for 5 min, and were rinsed again with PBS. Image acquisition was performed using a Leica confocal microscope (Mica, Leica Microsystems, Wetzlar, Germany), and visualization was achieved by applying the maximum intensity projection algorithm.

### 4.10. In Silico Investigation

UALCAN is a comprehensive, user-friendly, and interactive web resource for analyzing cancer OMICS data [33]. It is built to provide easy access to publicly available cancer OMICS data (accessed on 23 February 2024). We used it to provide graphs and plots depicting CBX1-8 expression profiles between normal and colon cancer tissues and according to cancer stage. The statistical significance is estimated by Student’s *t*-test and setting *p*-value < 0.05.

### 4.11. Statistical Analysis

Statistical analysis was conducted on both biological (N) and technical replicates (n). All data presented herein represent mean ± standard deviation (SD). Comparisons between controls and treated groups were performed using non-parametric statistical tests (Mann–Whitney U or Wilcoxon signed-rank tests and Kruskal–Wallis one-way ANOVA). All data were analyzed using Prism software 10.2.3 (Graph pad, Inc.). Differences were considered statistically significant for *p*-values below 0.05.

## Data Availability

The data presented in this study are available from the corresponding author upon request.

## Supplementary Materials

The following supporting information can be downloaded at https://www.mdpi.com/article/10.3390/ijms26157385/s1.
