# Supplementary material for: The Study of Chromobox Protein Homolog 4 in 3D Organoid Models of Colon Cancer as a Potential Predictive Marker
Source: Int J Mol Sci. 2025 Jul 30;26(15):7385. doi: 10.3390/ijms26157385 (PMC12347211; doi:10.3390/ijms26157385)
Supplement: Supplementary file 1 [file ijms-26-07385-s001.zip › Supplementary Figures.pdf]

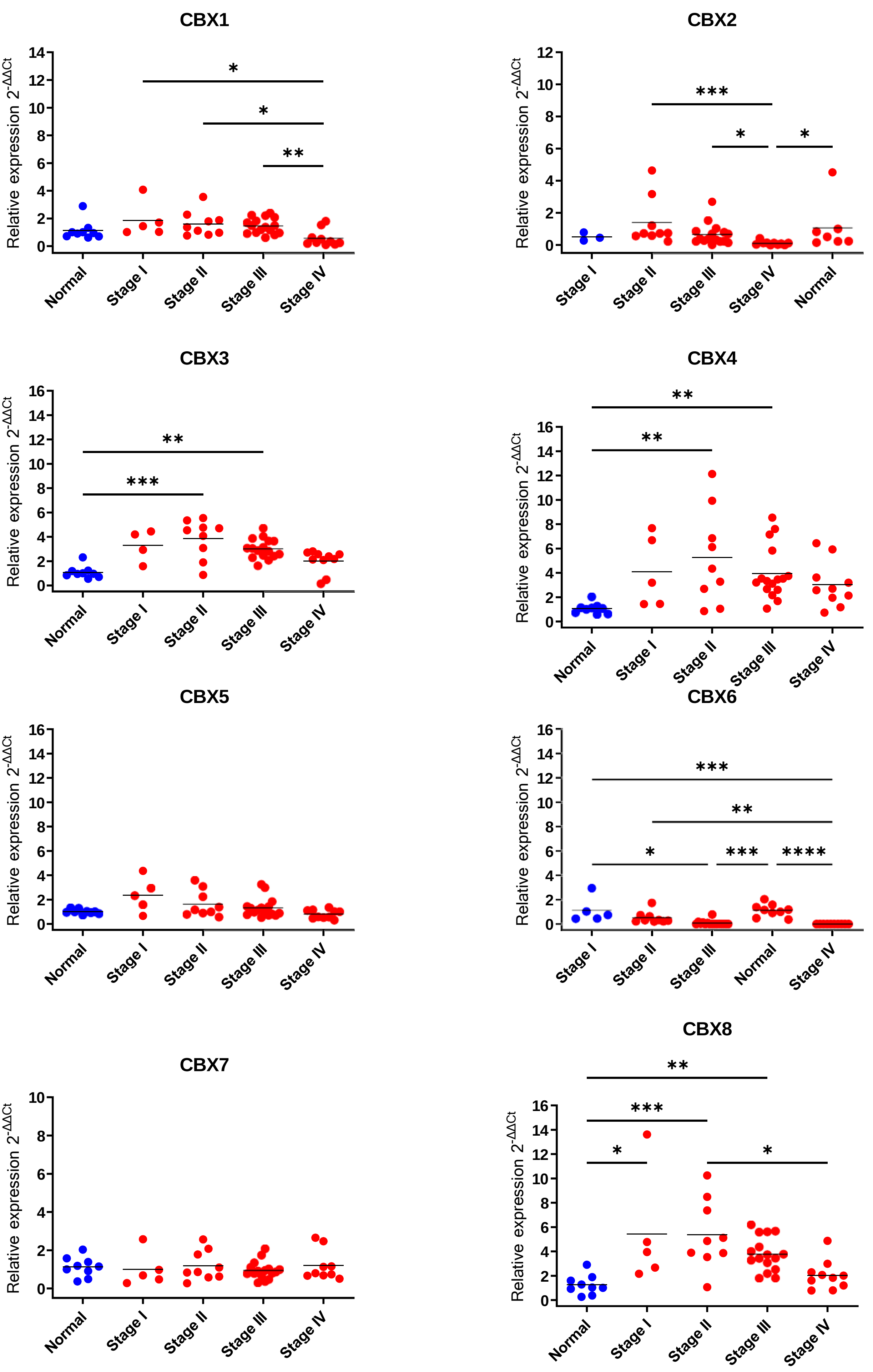

Supplementary Figure S1. CBX expression analyses in a colon cancer cDNA RT-qPCR array consisting of tumor (n=40) and normal samples (n=8) stratifying patients according to stage.

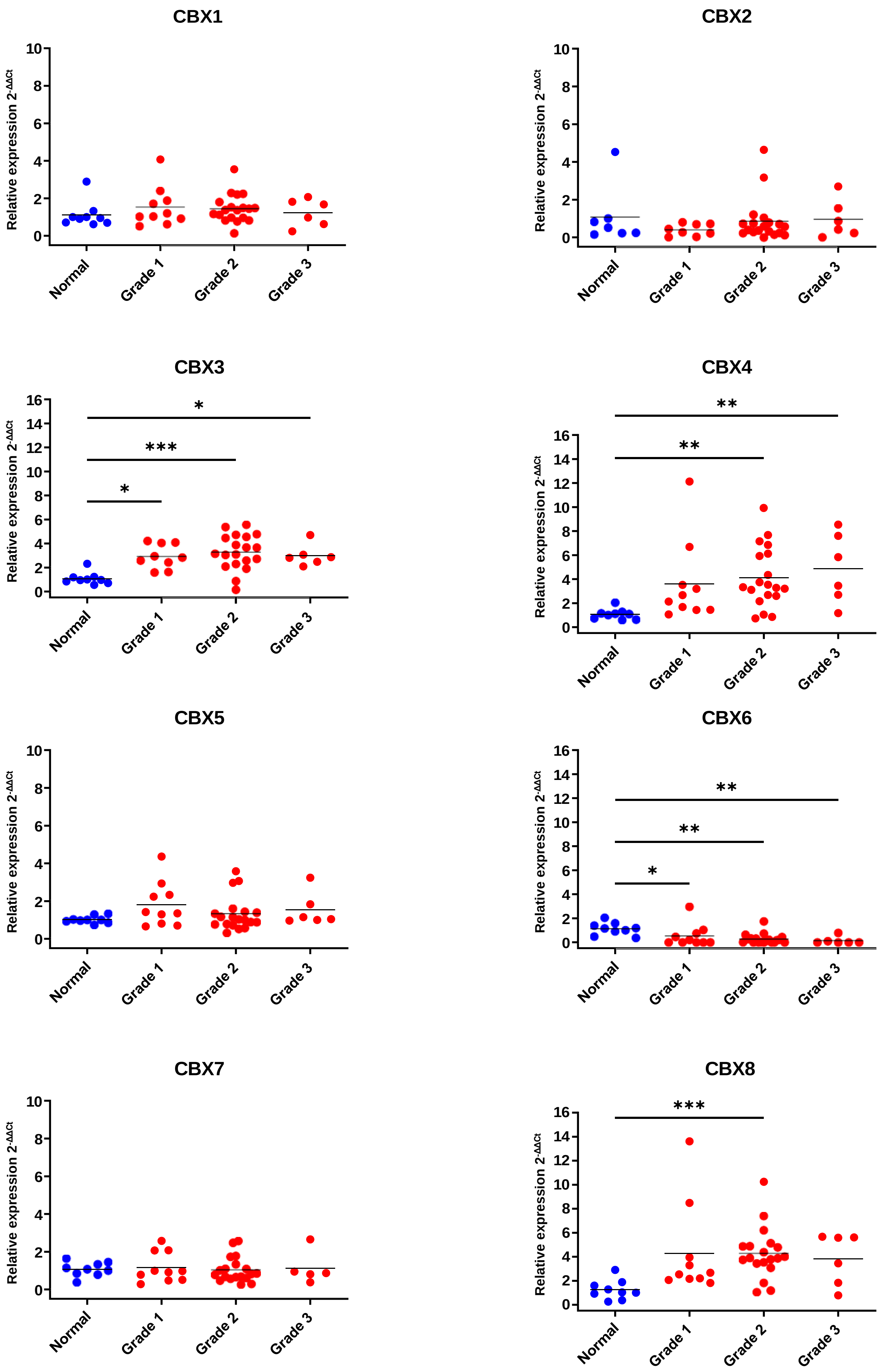

Supplementary Figure S2. CBX expression analyses in a colon cancer cDNA RT-qPCR array consisting of tumor (n=40) and normal samples (n=8) stratifying patients according to and grade.

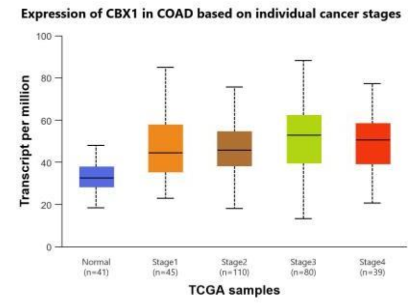

Comparison	Statistical significance
Normal-vs-Stage1	9.8608999998806E-07
Normal-vs-Stage2	4.93605156748345E-12
Normal-vs-Stage3	1.63791202822949E-12
Normal-vs-Stage4	5.61480000049741E-07
Stage1-vs-Stage2	6.993400E-01
Stage1-vs-Stage3	1.028630E-01
Stage1-vs-Stage4	3.704200E-01
Stage2-vs-Stage3	7.998800E-03
Stage2-vs-Stage4	1.771000E-01
Stage3-vs-Stage4	6.269000E-01

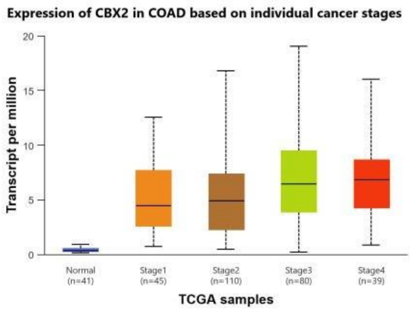

Comparison	Statistical significance
Normal-vs-Stage1	4.34240421398613E-11
Normal-vs-Stage2	<1E-12
Normal-vs-Stage3	1.62447832963153E-12
Normal-vs-Stage4	4.48500014904596E-10
Stage1-vs-Stage2	8.776000E-01
Stage1-vs-Stage3	2.166200E-02
Stage1-vs-Stage4	8.720100E-02
Stage2-vs-Stage3	1.304010E-02
Stage2-vs-Stage4	7.669900E-02
Stage3-vs-Stage4	7.920800E-01

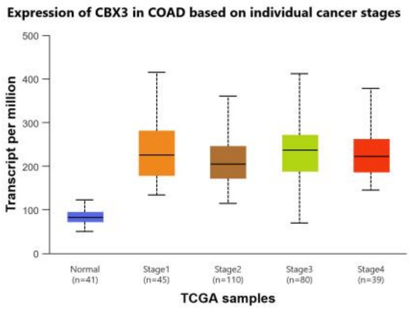

Comparison	Statistical significance
Normal-vs-Stage1	1.62458935193399E-12
Normal-vs-Stage2	1.62447832963153E-12
Normal-vs-Stage3	1.62447832963153E-12
Normal-vs-Stage4	1.62447832963153E-12
Stage1-vs-Stage2	1.141580E-01
Stage1-vs-Stage3	7.573000E-01
Stage1-vs-Stage4	7.721200E-01
Stage2-vs-Stage3	3.464000E-02
Stage2-vs-Stage4	2.208800E-01
Stage3-vs-Stage4	5.094600E-01

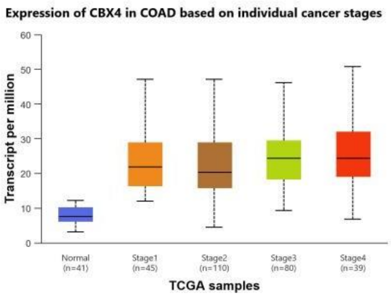

Comparison	Statistical significance
Normal-vs-Stage1	2.22044604925031E-16
Normal-vs-Stage2	<1E-12
Normal-vs-Stage3	<1E-12
Normal-vs-Stage4	5.15187892347058E-12
Stage1-vs-Stage2	7.296600E-01
Stage1-vs-Stage3	5.315800E-02
Stage1-vs-Stage4	8.700300E-02
Stage2-vs-Stage3	7.856000E-02
Stage2-vs-Stage4	9.997800E-02
Stage3-vs-Stage4	7.942200E-01

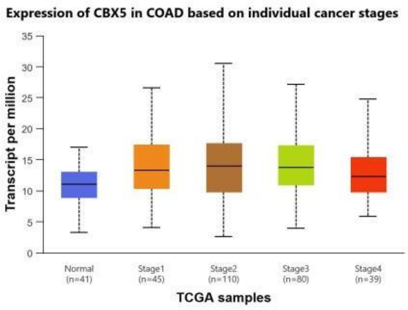

Comparison	Statistical significance
Normal-vs-Stage1	3.229900E-04
Normal-vs-Stage2	1.08369999995972E-06
Normal-vs-Stage3	4.93699999992714E-07
Normal-vs-Stage4	4.379700E-03
Stage1-vs-Stage2	5.296000E-01
Stage1-vs-Stage3	3.200600E-01
Stage1-vs-Stage4	7.817400E-01
Stage2-vs-Stage3	6.191400E-01
Stage2-vs-Stage4	4.337400E-01
Stage3-vs-Stage4	2.375200E-01

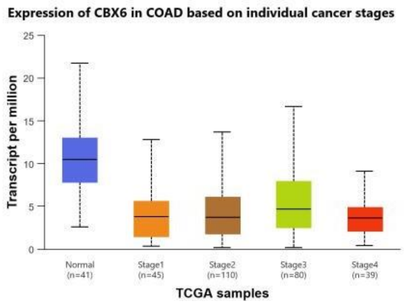

Comparison	Statistical significance
Normal-vs-Stage1	3.376400E-04
Normal-vs-Stage2	5.228900E-04
Normal-vs-Stage3	1.498210E-03
Normal-vs-Stage4	5.72079999999753E-05
Stage1-vs-Stage2	6.766400E-01
Stage1-vs-Stage3	2.995000E-01
Stage1-vs-Stage4	4.655800E-01
Stage2-vs-Stage3	5.097000E-01
Stage2-vs-Stage4	1.717630E-01
Stage3-vs-Stage4	4.161800E-02

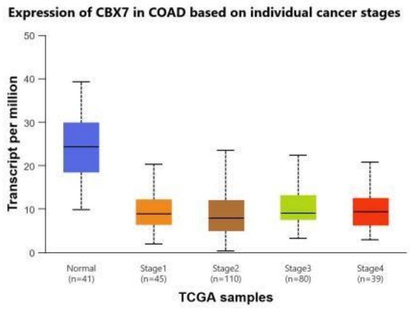

Comparison	Statistical significance
Normal-vs-Stage1	1.13200000484426E-08
Normal-vs-Stage2	6.75089995016975E-09
Normal-vs-Stage3	9.77209999586393E-08
Normal-vs-Stage4	6.02109999636724E-08
Stage1-vs-Stage2	6.462200E-01
Stage1-vs-Stage3	9.945700E-02
Stage1-vs-Stage4	3.419800E-01
Stage2-vs-Stage3	3.328200E-02
Stage2-vs-Stage4	1.706360E-01
Stage3-vs-Stage4	6.684200E-01

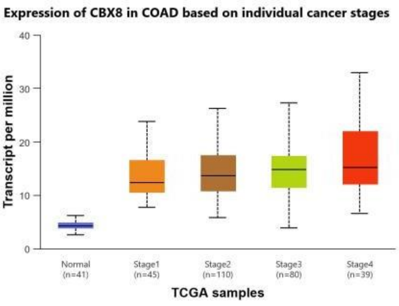

Comparison	Statistical significance
Normal-vs-Stage1	1.62458935193399E-12
Normal-vs-Stage2	1.62447832963153E-12
Normal-vs-Stage3	1.62447832963153E-12
Normal-vs-Stage4	1.64812608005604E-12
Stage1-vs-Stage2	6.883800E-01
Stage1-vs-Stage3	7.074300E-02
Stage1-vs-Stage4	5.114000E-02
Stage2-vs-Stage3	8.178300E-02
Stage2-vs-Stage4	6.797800E-02
Stage3-vs-Stage4	7.651200E-01

Supplementary Figure S3. Association between CBX expression levels and clinical stage of CRC patients. The CBX expression analyses performed using the UALCAN data set with relative statistical comparison among groups.

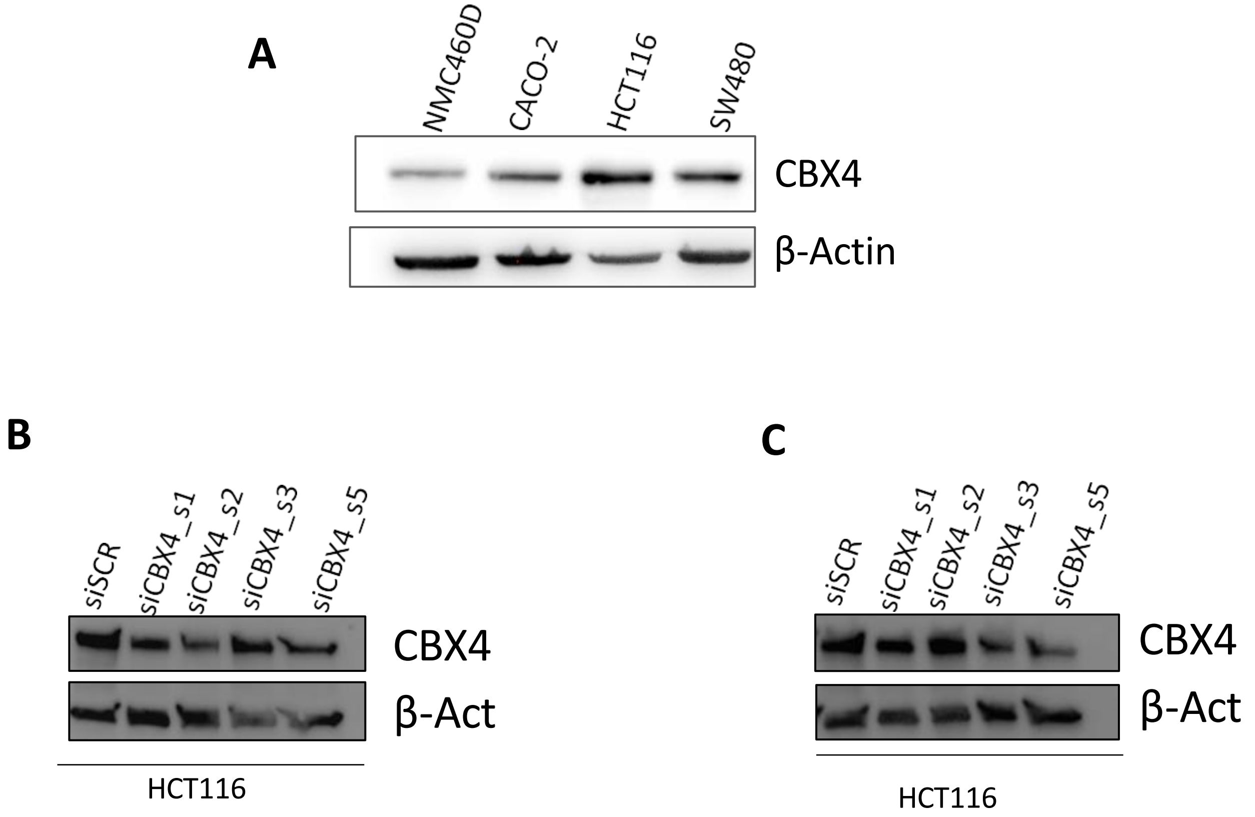

Supplementary Figure S4. CBX4 expression level by western-blot in three colon cancer cell lines (CACO-2, HCT116 and SW480) compared to normal colon cell line (NMC460D). B-C. Analysis of the knockdown efficiency of four sequences of siCBX4 (siCBX4_s1, siCBX4_ s2, siCBX4_ s3, siCBX4_ s5) by western-blot in HCT116 cell line compared to the scrambled sequence (siSCR) after 48h (B) and after 72h (C).
