# Supplementary material for: The Study of Chromobox Protein Homolog 4 in 3D Organoid Models of Colon Cancer as a Potential Predictive Marker
Source: Int J Mol Sci. 2025 Jul 30;26(15):7385. doi: 10.3390/ijms26157385 (PMC12347211; doi:10.3390/ijms26157385)
Supplement: Supplementary file 1 [file ijms-26-07385-s001.zip › Supplementary Table.pdf]

Supplementary Materials

Table S1. List of primers used for the detection of reported genes in colon cancer cell lines and PDOs.

Gene	Forward sequence	Reverse sequence
RPS18	CGCCGCTAGAGGTGAAATTC	CTTTCGCTCTGGTCCGTCTT
Nf-kB	GCAGCACTACTTCTTGACCACC	TCTGCTCCTGAGCATTGACGTC
c-Myc	CCTGGTGCTCCATGAGGAGAC	CAGACTCTGACCTTTTGCCAGG
TNFα	AGCCCATGTTGTAGCAAACC	CCAAAGTAGACTGCCCAGAA
IL-1	GCTGATGGCCCTAAACAGATG	TTGCTGTAGTGGTGGTCGGA
CBX1	GAGCTACAGACTCCAGTGGAGA	GTAGGAATGCCACGTCAGCCTT
CBX2	GGAACATGAGAAGGAGGTGCAG	GAAGAGGAGGAACTGCTGGACT
CBX3	GCTGACAAACCAAGAGGATTTGC	CAGCACCAAGTCTGCCTCATCT
CBX4	GCTGCTGATCGCCTTCCAGAAC	TTGGAACGACGGGCAAAGGTAG
CBX5	GAGAAGTCAGAAAGTAACAAGAGG	GTTCCAGTCCTCTCTCAAAGCC
CBX6	AGAGGGAGCGTGAGCTGTATGG	GCTCGGCTTGACAGAGAAATGC
CBX7	AGGAAGAGAGGTCCGAAACCCA	AGAAGCAGAGCTTCTCCTTGCC
CBX8	AACATCCTGGATGCTCGCTTGC	TTTGAGGAGGAAGGTTTTGGGCT
